# Neuroticism Level and Life Satisfaction in Women Undergoing Breast Augmentation Surgery (a Preliminary Report)

**DOI:** 10.1007/s00266-019-01308-6

**Published:** 2019-02-06

**Authors:** Daniel Zaborski, Teresa Rzepa, Maciej Pastucha, Andrzej Modrzejewski, Wilhelm Grzesiak

**Affiliations:** 1 Laboratory of Biostatistics, West Pomeranian University of Technology, Klemensa Janickiego 29, 71-270 Szczecin, Poland; 2 Department of General Psychology and History of Psychology, The Warsaw School of Social Sciences and Humanities, Poznan, Poland; 3 Private Medical Practice in the Field of Cosmetic Surgery, Szczecin, Poland; 4 Laboratory of Surgical and Emergency Nursing, Pomeranian Medical University, Szczecin, Poland

**Keywords:** Breast, Neuroticism, Plastic surgery, Quality of life

## Abstract

**Background:**

The aim of the present study was to verify the relationship between the level of neuroticism and selected aspects of life satisfaction in women undergoing breast augmentation surgery.

**Methods:**

The study group included 109 women, aged 18–46 years, who completed the self-developed survey measuring selected psychological traits before and after (1 year) surgery. Four questions in this survey were related to the level of neuroticism and two pertained to the self-assessment of leading character traits and the level of life satisfaction. Three questions made up the lie scale.

**Results:**

The studied women were constant in their truthfulness. No statistically significant difference in the level of neuroticism before and after surgery was noticed. However, an increase in the subjective life satisfaction after surgery was highly significant (*p* < 0.001). Statistically significant negative correlations of neuroticism level with the self-assessment of positive character traits (*r*_s_ = − 0.236; *p* = 0.013) and life satisfaction (*r*_s_ = − 0.277; *p* = 0.004) were found before surgery. Also, a significant positive correlation was observed between neuroticism and the change in life satisfaction 1 year after surgery (*r*_s_ = 0.302; *p* = 0.001).

**Conclusions:**

Breast augmentation surgery did not affect neuroticism level, which proves that constitutional personality traits in women undergoing such operations are not significantly influenced by a surgical intervention. However, neuroticism may play the role of a modulator of the psychological changes in women after breast augmentation (e.g., an increased postsurgical life satisfaction). The preliminary results obtained in our study should be confirmed on a larger sample size in the future.

**Level of Evidence IV:**

This journal requires that authors assign a level of evidence to each article. For a full description of these Evidence-Based Medicine ratings, please refer to the Table of Contents or the online Instructions to Authors www.springer.com/00266.

## Introduction

According to many previous studies [1–4], a typical patient undergoing breast augmentation surgery is most frequently Caucasian, slim, tall, well-educated, aged 28–44 years, smoking and drinking alcohol, with an increased chance of depressive and anxiety states and neurotic personality [5, 6]. Neuroticism as an element and determinant of temperament type [7] is linked to a tendency to react in an inadequate and most often excessive way, especially in stressful situations [8]. This is associated with the implementation of maladaptive strategies of coping with stress such as behavioral disengagement and helplessness [9], which is often accompanied by strong negative emotions such as anxiety, fear, irritability, irritation, and sadness. Apart from excessive negative emotionality, neurotic persons show a tendency to perceive the world as a dangerous, threatening and hostile place. They become convinced of their inability to cope with life’s challenges and interpersonal relationships. These traits and dispositions constitute the so-called negative triad [10]. Neuroticism can therefore be defined as a permanent disposition to feel predominantly negative emotions associated with a sense of insufficient control over the course of one’s life [11].

Neuroticism also belongs to the Big Five personality traits (also known as the five-factor model), i.e., five broad domains or dimensions (neuroticism, extraversion, openness, altruism, and conscientiousness) that are used to describe human personality. The Big Five model is one of the most widely used, validated and well-known psychological models of personality. The examples of the factors evaluated in it are as follows: experience of psychological distress (Neuroticism; N), experience of positive emotions, sociability, talkativeness, and energy (Extraversion; E), sensitivity to art, imagination, intellectual curiosity, and behavioral flexibility (Openness to Experience; O), trust, cooperativeness, and sympathizing with others (Agreeableness; A), morality, organization, and diligence (Conscientiousness; C). In general, the higher levels of Extraversion, Openness to Experience, Conscientiousness, and Agreeableness seem to be positively related to resilience, health-related quality of life and relationship satisfaction, whereas a higher level of neuroticism has a negative effect on them [12]. The above-mentioned factors can be evaluated with, e.g., the Neuroticism–Extraversion–Openness to New Experience Five-Factor Inventory (NEO-FFI) [13, 14].

In previous studies, an association has been found between the occurrence of neuroticism and a tendency to undergo aesthetic surgery (including breast augmentation) [15]. For instance, patients undergoing rhinoplasty were assessed using the Maudsley Personality Inventory (MPI). After surgery, they showed decreased neuroticism and increased extraversion [16, 17]. Groenman [18] examined 25 patients undergoing breast augmentation who did not present preoperative neurotic symptoms. The cited author used the same inventory (MPI) and found that a postoperative tendency toward neurotic states (anxiety, internal tensions, fear) was reduced along with the proneness to neurotic behavior such as complaints about somatic symptoms (sweating, tiredness, sleep problems, headaches, or extensive heartbeat).

Cosmetic surgery plays an important role in patient life satisfaction. As stated by Biggs et al. [19] more than 35 years ago: “The purpose of all aesthetic surgery is to improve the quality of life of the patient through an enhancement of her or his own self-image.” According to Barone et al. [20] candidates from different occupational or educational groups expect an improvement in their quality of life after a cosmetic surgery procedure. However, those with a lower educational level usually have less realistic expectations about the postoperative outcomes. The still-increasing frequency of cosmetic operations performed each year worldwide results from the patients’ search for a state of well-being partially based on their physical appearance. Pursuing beauty is not only associated with an aesthetic ideal but also with attaining one’s own life satisfaction [21]. It has been altered by the modified concept of health, which changed from simply being the absence of a disease into an idea of physical, psychological and social well-being [22]. To be more specific, several studies [23–26] have shown an improved quality of life, better psychosocial and sexual well-being as well as greater satisfaction with breast appearance after their augmentation. Also, the more recent application of the validated Breast-Q questionnaire to the assessment of psychosocial well-being, among others, showed a significant increase in its mean score after breast augmentation surgery [27]. A rise in self-esteem, satisfaction with one’s own appearance and interpersonal confidence, was observed in patients undergoing this type of cosmetic operation [28]. In addition, the study on patients’ satisfaction using a self-developed questionnaire [29] revealed a greater motivation for performing everyday activities, a more intense feeling of being a “whole” person and better social competencies. The authors of the cited study also reported that the majority of women (about 93%) were satisfied with the outcomes of the surgery. A similar work [30] on the psychological benefits of breast augmentation for aesthetic purposes showed that body image and self-esteem improved postsurgery, whereas depressive symptoms subsided. A higher level of satisfaction with one’s breasts, psychosocial and sexual well-being was noticed in patients evaluated before surgery and two times after using the Breast-Q questionnaire. The aforementioned results were confirmed in the review on life satisfaction in persons undergoing different types of cosmetic surgery. It showed that the candidates do not only expect positive changes in their appearance but also those in health-related quality of life. In general, the overall quality of life in patients before surgery is usually worse compared with the control group. Moreover, an improvement in life quality increases until about 1 year postsurgery. Conversely, such changes are not so evident later on [31].

Due to the relatively small number of previous scientific reports, the aim of the present study was to verify the relationship between the level of neuroticism and selected aspects of life satisfaction in women undergoing breast augmentation surgery.

## Materials and Methods

**Fig. 1:**
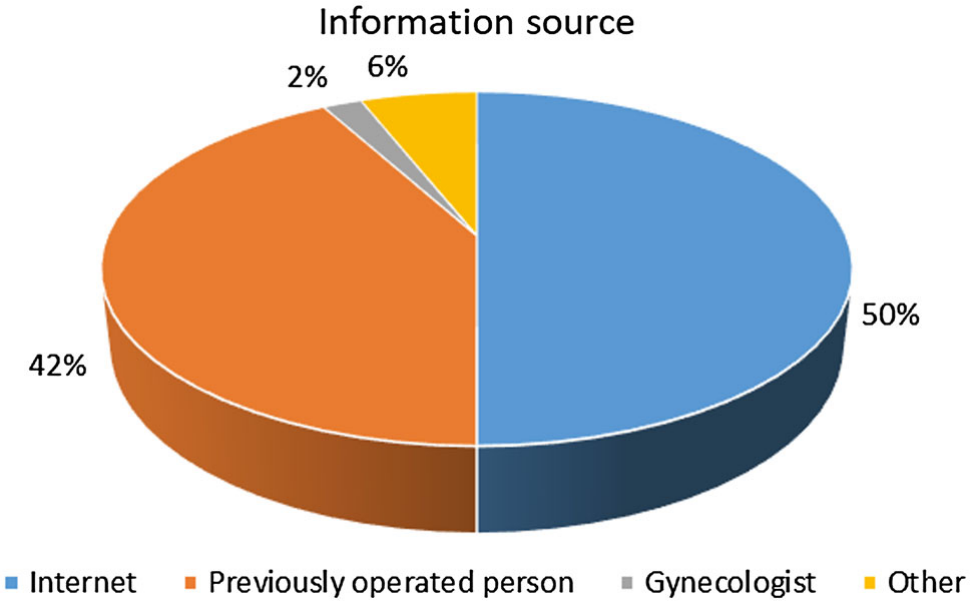
The source of information about breast augmentation surgery

The study was carried out between 2008 and 2015. The protocol has been approved by the Bioethics Committee of the Medical University (Approval No. KB-0012/261/07/18). The study group included 109 women who underwent breast augmentation surgery in a private medical practice. There were no specific inclusion and exclusion criteria for the study. All women eligible for the surgery (consecutive patients) who declared that they would complete the survey before and after the procedure were requested to participate in the study. The women’s mean age was 30.7 years (SD = 5.4 years, min = 18 years, max = 46 years; Table 1). They were mainly married or single and lived in rather large towns (above 50,000 residents). The women found out about the place and possibility of surgery mainly from the Internet (50%), previously operated persons (42%), or gynecologists (2%). Approximately 6% of patients gained information from other sources (Fig. 1).

**Table 1:** Patient demographics (*n* = 109)

Variable	Mean	SD
Age (years)	30.7	5.4

	*n*	%
Marital status		
Single	44	40.37
Married	57	52.29
Widow/divorced	8	7.34
Population of the town where the patients lived		
< 10,000	25	22.94
10,000–50,000	25	22.94
> 50,000	59	54.13

A self-constructed survey, consisting of 16 different questions measuring selected sociodemographic and psychological variables, was used in a two-stage, preliminary study. Four questions were related to the level of neuroticism (scored from 0 to 2), three others made up the so-called lie scale measuring social honesty (scored from 0 to 2), and two pertained to the assessment of leading character traits (a maximum of five items selected from the list including positive and negative adjectives describing the patient’s character) and the subjective level of life satisfaction (scored from 0 to 10). The survey is presented in Table 2 and has been successfully applied in our previous study [32].

**Table 2:** The survey used in the study

Dimension	Contents		
Questions present in the original survey and not used in the present study	The most important events in my life (select up to 3 items): Acquiring profession, marriage, childbirth, death of a close person, change of a living place, first love, purchase of a flat, first job, decision on altering my breasts, leaving family house, other		
	The most important values to me (select up to 2 items): Money, health, professional success, happiness of a close person, love, my own appearance, successful family life, social success, other		
	How will the decision on surgery change my life (select 1 item)?^a^ It will entirely change my life. It will mainly change my sexual life. It will change my self-image into positive. It will not affect me at all. It is insignificant because I have high self-assessment anyway. Other.		
	How has the decision on surgery changed my life (select 1 item)?^b^ It has entirely changed my life. It has mainly changed my sexual life. It has changed my self-image into positive. It has not affected me at all. It has been insignificant because I have high self-assessment anyway. I have stopped envying other women their appearance. Finally, I undress on the beach without inhibitions. I have become more attractive for my partner. Other.		
	I have learnt about the place and possibility of surgery from (select 1 item):^a^ The poster at the doctor’s surgery. “Info-Tip” journal published in Germany. The leaflet at the restaurant in Poland. The previously operated person. “The Polish Courier” newspaper published in Germany. The gynecologist. The hair-dresser, who heard about such a possibility. The Internet. Other.		
	The reason for deciding on cosmetic surgery (select up to 2 items):^a^ It is a matter of my physical and mental state. I do not feel good with my breasts. I am ashamed of my breasts. I have a problem with undressing on the beach. I am ashamed of my breasts. I have difficulties in undressing in my partner’s presence. My breasts are my inveterate complex. I want to get rid of it. I am doing it out of envy. I cannot look at women with perfect breasts. It is not only a matter of breasts. My appearance is important to me. My present breasts are too small. I have always wanted to have large breasts. I just feel like improving my appearance. I am doing it for my partner, who wants me to have proper breasts. Small breasts make my sexual life difficult. I have made a decision on breast augmentation together with my partner. Other.		
	Assessment of my appearance (select 1 item): Very attractive, without any fault. Rather attractive, certainly I favorably stand out from the crowd. Rather average, not different from others. I still have a lot of objections to my appearance. I am not attractive. I am going to undergo another cosmetic surgery. Other.		
	How do I deal with difficult situations (select up to 2 items)?^a^ I just try not to think about it. I ask somebody’s advice on what to do. I fall asleep and believe that the problem will disappear when I wake up. I confide in somebody but do not expect any advice. I try to solve the problem by myself as quickly as possible. I usually sit and worry myself. I eat more than usually—I just “overeat” my trouble. I stop eating then. I smoke more than usual. When I worry I believe in the effectiveness of alcohol. Other.		
Personality traits	The following adjectives describe me best (select up to 5 items): Intelligent, hard-working, beautiful, ugly, impatient, lost and confused, nervous, frank, witty, lonely, responsible, happy, active, calm, grumbling, nice, tidy, nasty, caring, lazy		
Quality of life assessment	Current satisfaction with my life (0—lowest, 10—highest)?		
Neuroticism	Something has troubled me for a long time, as if something bad was going to happen:		
	Yes	No	Difficult to say
	In my opinion, you cannot trust even the loved ones, because I have been often disappointed in them:		
	Yes	No	Difficult to say
	I often worry about some trifles that generally should not bother me:		
	Yes	No	Difficult to say
	I prefer keeping away from others because many people wish me ill:		
	Yes	No	Difficult to say
Lie scale	I sometimes boast:		
	Yes	No	Difficult to say
	I sometimes put off things I should do today until tomorrow:		
	Yes	No	Difficult to say
	I sometimes lie:		
	Yes	No	Difficult to say

^a^Questions asked only before surgery

^b^Questions asked only after surgery

All women scheduled for surgery who declared that they would complete the survey before and after the procedure were included in the study. The survey was filled out by the patients while waiting for their medical appointment: for the first time, before the breast augmentation surgery (190 completed copies) and for the second time, 1 year after surgery, just prior to the follow-up visit (115 completed copies). Out of 115 copies filled out before and after surgery, 109 were complete. For both measurements, the lie scale values indicated the general truthfulness of the studied women.

The structure of the lie scale was based on a similar principle as the structure of such scales in questionnaires and psychological tests—on the self-description of the common social behavior (e.g., I sometimes boast, I sometimes put off things I should do today until tomorrow). If a respondent provides more answers contradicting the occurrence of such behavior in her case, then there is a suspicion that these answers can be distorted, which is associated with a tendency to distort the answers to other questions as well.

The sample size used in the present study was not calculated before its execution. All available data were used for the analysis. The survey results were verified statistically. The Wilcoxon signed-rank test was applied to determine differences in the level of psychological traits before and after surgery [33]. Additionally, Spearman’s rank correlation coefficient (*r*_s_) was used to analyze the relationship of neuroticism levels with selected character traits, lie scale and life satisfaction [34]. In order to calculate the *r*_s_ coefficients, the values of a given variable were first converted from the nominal (answers to individual questions) to the rank scale. Statistical analysis was carried out using Statistica software (v. 12, StatSoft Inc., Tulsa, OK, USA). Statistical significance was declared at *p* ≤ 0.05. Values on the lie and neurotic scales as well as life satisfaction are presented as means and standard deviations.

## Results

An analysis of the responses to questions pertaining to the lie scale showed that the studied women were constant in their truthfulness (a nonsignificant difference in the values on the lie scale before and after surgery, *Z* = 1.476, *p* = 0.140, Table 3). Analyzing the sum of four questions related to neuroticism, no statistically significant difference in the level of this personality disposition before and after surgery was observed (*Z* = 1.301; *p* = 0.193; Table 3). However, there was a noticeable difference in the assessment of the first neurotic indicator: After the surgery, patients were less likely to declare unspecific fears about anticipated negative events that may happen in the future (Z = − 3.074; *p* = 0.002; Table 3). An increase in the subjective life satisfaction after surgery was highly significant (*Z* = 5.706; *p* < 0.001; Table 3).

**Table 3:** The mean ranks of neuroticism, lie scale and life satisfaction before and after breast augmentation

Variable	Before			After		
	Range	Mean	SD	Range	Mean	SD
Neuroticism	0–8	2.58	1.87	0–8	2.40	1.62
Lie scale	0–6	2.34	1.61	0–6	2.19	1.68
Life satisfaction	1–10	7.62**	1.67	5–10	8.37	1.35

**Statistical significance at *p* ≤ 0.01

There were no significant associations between neuroticism and lie scale as well as the level of neuroticism and the remaining variables analyzed postsurgery. A statistically significant correlation was observed between the level of neuroticism and patient’s self-assessment of character traits and life satisfaction before surgery. As shown in Table 4, women with a higher level of neuroticism selected less positive character items to describe themselves (*p* = 0.013) and declared a lower life satisfaction (*p* = 0.004). However, such a relationship was not observed 1 year after operation. Additionally, it was found that the difference in life satisfaction before and after the surgery was correlated with the initial level of neuroticism (*r*_s_ = 0.302; *p* = 0.001), which means that an improvement in life satisfaction increased with an increasing level of neuroticism before surgery. It should also be emphasized that all of the observed correlations were generally weak, though in some cases statistically significant. None of the tested variables correlated significantly with the age of the participating women.

**Table 4:** The Spearman rank correlation coefficients between the neuroticism level measured before surgery and selected traits before and after surgery

Trait	Before	After
Assessment of character traits		
Positive adjectives	− 0.236*	− 0.091
Negative adjectives	0.115	0.118
Lie scale	− 0.055	− 0.111
Life satisfaction	− 0.277**	− 0.116

*Statistical significance at *p* ≤ 0.05; **p ≤ 0.01

## Discussion

The present study showed that breast augmentation surgery did not affect the neuroticism levels, which were assessed before surgery and 1 year postsurgery. The mean value of this variable was lower after surgery, but it did not differ significantly from that before the operation. This finding seems justified since, as described in the introduction, neuroticism is a personality trait, which is quite constant in adult women, although some changes may occur in younger patients, below approximately 25 years of age [35]. It should, however, be taken into account that the objective assessment of this trait may be difficult under some circumstances, as the outcome of such evaluation may be partially dependent upon the current life situation of the examined person [36].

A statistically significant negative correlation between the levels of neuroticism and life satisfaction in women examined before breast cosmetic surgery was observed in our study. Hence, a conclusion can be drawn with some caution that women with a higher neuroticism level before operation were characterized by lower life satisfaction compared with their less neurotic counterparts. Moreover, surgery had a beneficial effect on their subjective evaluation of the overall quality of life, which markedly improved 1 year after surgery.

The effect of breast augmentation on the self-reported quality of life has already been described in literature. A scale similar to the one in this study was used by Swanson [37] to assess the overall satisfaction after breast augmentation. He recorded a mean value of 9.3 points, which was higher than that (8.4 points) obtained in our study. However, Swanson did not report the initial level of satisfaction (before surgery), so it is difficult to refer this result to its baseline value. The same study also revealed a significant improvement in the self-perceived quality of life—one-third of the examined women declared considerable improvement, one-third slight improvement and the same proportion of patients a lack of improvement [37]. Sarwer et al. [38], using the Body Image Quality of Life Inventory (BIQLI), which assesses the effect of body image on the overall quality of life, did not observe any increase in its level. However, several other aspects such as body image improved when evaluated 3-, 6- and 12-months postsurgery. Also, the dissatisfaction with the appearance of the operated body part was lower and the feeling of negative emotions associated with appearance was less frequent. The next similar study [39] performed using the self-developed survey revealed the lack of significant improvement in the broad aspects of life quality such as physical health, activity, or working capacity, although substantial positive changes in body perception, attractiveness and femininity were observed. In later work on the long-term effects of the Style 410 silicone breast implants [40], the lack of significant changes in the overall quality of life (physical health, activity and working capacity) was shown. The only general aspect of life quality that improved in women undergoing breast augmentation (70% of all examined patients) was their overall well-being.

The subsequent study on the long-term effects of breast augmentation using the Natrell breast implants did not show any increase in health-related quality of life, although an improvement in body image was noticed. Due to the lack of appropriate psychometric tools for the assessment of the outcomes of breast cosmetic surgery from the patient’s perspective, Pusic et al. [41] developed a new Breast-Q questionnaire in 2009, which consists of three main modules (applied both before and after surgery) for breast augmentation, reduction, and reconstruction. The modules were developed to assess the patient’s satisfaction with the performed surgery and the changes in the health-related quality of life. In the study [24] on the satisfaction of breast augmentation patients and health-related quality of life using the above-mentioned questionnaire completed once before surgery and once 2 months after, significant changes in the level of satisfaction with one’s breasts, psychosocial and sexual well-being were found. The next work on the satisfaction and well-being in women undergoing breast augmentation surgery evaluated using the Breast-Q questionnaire [25] applied once before surgery and once 6 weeks after, showed a significant increase in the satisfaction with one’s breasts, psychosocial and sexual well-being, as well as an opposite trend in physical well-being, which substantially decreased after the operation. The overall satisfaction with the outcome of the surgery was most strongly correlated with breast appearance, and to a lesser extent, with psychosocial or sexual well-being. No significant correlation was found between the overall satisfaction and physical well-being. The above-mentioned results were confirmed on a larger sample of patients surveyed three times (once before surgery, once 6 weeks after and once 6 months after). A significant increase in satisfaction with one’s breasts, psychosocial and sexual well-being was observed. No significant change in physical well-being was found, which was even lower after surgery than before.

In the cited study, a significant relationship was observed between the patient’s age and the overall satisfaction with the results of the surgery (older women were less satisfied). It should be mentioned that no significant correlations were found between the patient’s age and life satisfaction in the present study. The final relationship reported in the cited article was found between the overall satisfaction with the surgery and postsurgical improvement in the quality of life and breast implant type. However, such a relationship was not analyzed in our study. Finally, a more recent work [42] on the improvement in the quality of life in Asian women undergoing breast augmentation surgery, measured using the Breast-Q questionnaire, revealed a significant increase in the quality of life after surgery. The general satisfaction with the surgery outcomes was high, with a particular increase in the psychosocial and sexual well-being.

It should be emphasized that, besides Breast-Q, other questionnaires are also used for the evaluation of postsurgical quality of life. For instance, the European Quality of Life-5 Dimensions (EQ-5D) questionnaire consisting of items pertaining to pain, mobility, mood, self-care, activity, sleep, sex, and analgesic usage was applied to the evaluation of patients undergoing the same-day breast augmentation surgery [43]. The assessment was performed before the operation and 1, 3 and 6 months after it and revealed a subjective postsurgical discomfort in 20% of the patients in the first month postsurgery, which was mainly caused by pain and difficulties in mobility. However, these symptoms subsided completely in 97% of women about half a year after surgery. The body image assessment questionnaire [44] was also used to evaluate attractiveness/self-confidence, fear/anxiety, attention paid to one’s physical appearance and sexual life discomfort before and after breast augmentation surgery. The study revealed a higher self-rating of attractiveness and self-confidence, increased sexual life satisfaction and a positive change in self-perceived physical appearance. Only the level of fear/anxiety remained almost invariant. Similar results were obtained by Saariniemi et al. [28], who applied the 15D health-related quality of life questionnaire, among others. They found no significant difference in the overall quality of life before and after surgery, although more intense (compared with the general population) sleeping problems persisted before operation. However, some considerable improvements in the more detailed aspects of quality of life such as discomfort and symptoms, depression or vitality were observed. Only mental health deteriorated.

In the present study, a weak but statistically significant, negative correlation between the neuroticism level and a positive assessment of character traits was found in the women before breast augmentation surgery. Previous findings have shown that neuroticism is an important temperamental trait since neurotic persons are emotionally unbalanced and diffident. Usually, they have a low self-evaluation and tend to experience negative emotional states (anxiety, sadness, shame, and guilt). They strongly react to stressful situations and social assessments, experiencing a fear of getting hurt, punishment and losing control, which manifests itself in frequent mood changes and depressive disturbances [45]. Hence, they are usually perceived as capricious and depressed persons, blaming themselves for their own behaviors, even for those beyond their control. This, in turn, leads them to worry and be subject to depressive states, which are accompanied by complaints about insomnia and somatic ailments [7, 10, 46, 47]. It is known from previous studies that the level of neuroticism in patients suffering from depression is statistically significantly higher than that in healthy subjects [48, 49]. It was also shown that the presence of neuroticism is an indicator of susceptibility to depression developing as a consequence of stressful life events [50, 51]. Moreover, it was found that excessive self-criticism and oversensitivity, also due to one’s own physical appearance, reduce self-evaluation and self-esteem in neurotics [46].

It should be emphasized that neuroticism is a stable personality trait that reflects a chronic tendency to experience negative emotions and to stress reactivity. It is also a strong predictor of depressive illness, since it shares a genetic liability with major depression [52]. Neuroticism is closely tied to the temperament of negative affectivity and linked to Gray’s behavioral inhibition and activation systems that motivate withdrawal and approach behavior [36]. Neuroticism as a trait (belonging to the Big Five personality traits, also known as the five-factor model) encompasses a broad spectrum of heterogeneous traits, which are hierarchically organized into lower-level traits, called facets (Anxiety, Angry Hostility, Depression, Self-Consciousness, Impulsiveness, and Vulnerability) [53]. They are considered to be heritable and highly stable in adulthood [52]. In addition, each of them may be associated with the emotional and behavioral aspects of psychiatric symptoms or mental diseases [53]. However, it should be noticed that some studies [54, 55] have indicated the occurrence of mean-level changes in personality traits throughout the course of one’s life. Moreover, the assessment of temperament and personality is influenced by stable “trait” variance (usually being the target of evaluation) and transient “state” factors (such as current mood or situational influences), which makes it even more difficult to precisely determine the true changes in traits and the influence of current mood states [36].

The relationship between desire for cosmetic surgery and the presence of a neurotic personality has also been described in the literature. Pavan et al. [56] found that patients with body dysmorphic disorder seeking cosmetic surgery procedures were characterized by the higher Neo Five-Factor Inventory (NEO-FFI) scores for neuroticism and lower ones for extraversion, consciousness, and openness to experience, compared with the control group. Scharschmidt et al. [57] recorded the significantly higher scores of personality traits (extraversion, agreeableness, openness to experience, and neuroticism) and health-related quality of life in patients undergoing treatment with botulinum toxin A and dermal fillers. These and similar findings led us to verify the relationship between neuroticism and a tendency to undergo subsequent cosmetic surgeries in order to improve one’s physical appearance. It was also found that women intent on breast surgery were particularly dissatisfied with this part of their body [58, 59] and that patients set on such surgery had a higher probability of possessing traits typical of the neurotic personality [5]. In the present study, first of all, a significant dynamic relationship was noticed between the assessment of character traits and the level of neuroticism. Before surgery, women with a lower level of neuroticism were characterized by higher self-evaluation at the same time. Also, the relationship between neuroticism and life satisfaction is noteworthy. Although the correlation between them was low and nonsignificant after surgery, a significant positive correlation coefficient between the initial level of neuroticism and the difference in life satisfaction was observed, which means that the higher the level of neuroticism before surgery, the greater the improvement in life satisfaction. This result may confirm the beneficial effect of cosmetic surgery on patients with neurotic personality.

Finally, the following limitations of our study should be mentioned: the sample size was rather small (*n* = 109) taking into account the power of the test necessary to reject the null hypothesis. Therefore, the preliminary results presented in our study should be verified on a larger sample size. Secondly, no specific inclusion and exclusion criteria were applied in the recruitment procedure. Each patient declaring that she would complete the survey before and after surgery could have entered the study. Thirdly, no control group was included in the study, which limits to some extent the utility of conclusions drawn from it. Lastly, since it is known that a correlation exists between breast augmentation and suicide rate, the period of 1-year follow-up may not be long enough to observe real associations between the neuroticism level and life quality. Therefore, the future research should include a control group and the longer follow-up period to more accurately determine the relationships observed in the present work.

## Conclusions

It can be concluded that breast augmentation surgery did not affect the level of neuroticism. This shows that constitutional personality traits in women undergoing such operations are not significantly influenced by a surgical intervention. Personality traits are relatively stable constructs and remain at a similar level in adult persons, which is not affected by the cosmetic intervention into one’s own body. However, the results of the present study suggest that neuroticism (as a personality trait) may play the role of a modulator of the psychological changes in women after breast augmentation (e.g., an increased postsurgical life satisfaction), which is an interesting finding that should be further explored in future research. Taking into account the preliminary character of the present study, the obtained results should be confirmed on a larger sample size in the future.
